# Opening up safely: public health system requirements for ongoing COVID-19 management based on evaluation of Australia’s surveillance system performance

**DOI:** 10.1186/s12916-022-02344-x

**Published:** 2022-04-15

**Authors:** Kamalini Lokuge, Katina D’Onise, Emily Banks, Tatum Street, Sydney Jantos, Mohana Baptista, Kathryn Glass

**Affiliations:** 1 National Centre for Epidemiology and Population Health, The Australian National University, 62 Mills Road, Acton ACT, Canberra, 2601 Australia; 2 School of Public Health, University of Adelaide, Adelaide, Australia; 3 Wellbeing SA, Adelaide, South Australia Australia; 4 Department of Health, Melbourne, Victoria Australia

**Keywords:** COVID-19, SARS-CoV-2, Surveillance, Variants of concern, Detection, Community transmission, Genomic sequencing, Vaccination, Public health

## Abstract

**Background:**

Severe acute respiratory syndrome coronavirus 2 (SARS-CoV-2) community transmission was eliminated in Australia from 1/11/2020 to 30/6/2021, allowing evaluation of surveillance system performance in detecting novel outbreaks, including against variants of concern (VoCs). This paper aims to define system requirements for coronavirus disease 2019 (COVID-19) surveillance under future transmission and response scenarios, based on surveillance system performance to date.

**Methods:**

This study described and evaluated surveillance systems and epidemiological characteristics of novel outbreaks based on publicly available data, and assessed surveillance system sensitivity and timeliness in outbreak detection. These findings were integrated with analysis of other critical COVID-19 public health measures to establish future COVID-19 management requirements.

**Results:**

Twenty-five epidemiologically distinct outbreaks and five distinct clusters were identified in the study period, all linked through genomic sequencing to novel introductions from international travellers. Seventy percent (21/30) were detected through community testing of people with acute respiratory illness, and 30% (9/30) through quarantine screening. On average, 2.07% of the State population was tested in the week preceding detection for those identified through community surveillance. From 17/30 with publicly available data, the average time from seeding to detection was 4.9 days. One outbreak was preceded by unexpected positive wastewater results. Twenty of the 24 outbreaks in 2021 had publicly available sequencing data, all of which identified VoCs. A surveillance strategy for future VoCs similar to that used for detecting SARS-CoV-2 would require a 100–1000-fold increase in genomic sequencing capacity compared to the study period. Other essential requirements are maintaining outbreak response capacity and developing capacity to rapidly engineer, manufacture, and distribute variant vaccines at scale.

**Conclusions:**

Australia’s surveillance systems performed well in detecting novel introduction of SARS-CoV-2 while community transmission was eliminated; introductions were infrequent and case numbers were low. Detection relied on quarantine screening and community surveillance in symptomatic members of the general population, supported by comprehensive genomic sequencing. Once vaccine coverage is maximised, future COVID-19 control should shift to detection of SARS-CoV-2 VoCs, requiring maintenance of surveillance systems and testing all international arrivals, alongside greatly increased genomic sequencing capacity. Effective government support of localised public health response mechanisms and engagement of all sectors of the community is crucial to current and future COVID-19 management.

**Supplementary Information:**

The online version contains supplementary material available at 10.1186/s12916-022-02344-x.

## Background 

Countries recognised as having the best initial responses to the coronavirus disease 2019 (COVID-19) pandemic include Singapore, Taiwan, South Korea, Australia, New Zealand, Canada, Iceland, UAE, Germany, and Greece. These countries were largely successful in minimising morbidity and mortality prior to the availability of vaccines [1, 2]. Key to Australia’s success was early identification of community transmission through surveillance, control of outbreaks through non-pharmaceutical public health measures restricting mobility and social interaction, and effective management of international borders which reduced the risk of subsequent re-introductions [3, 4]. In addition to reducing health impacts, these measures have limited the number and duration of restrictions on community mobility and interaction. Consequently, the social and economic burdens of the pandemic were lower in Australia than in many other countries [5].

In the initial phases of the global pandemic, reliance on nationwide lockdowns in Australia was necessitated by delayed closure of international borders [6], which allowed community transmission to establish itself throughout many urban settings, and by limited surveillance, testing, and public health contact tracing capacity for detection and control of such transmission [7]. From November 2020 to June 2021, with improved capacity and capability of public health systems, and a reduction in the number of introductions from international arrivals, Australia was able to effectively identify and control outbreaks with a combination of measures, which sometimes included short lockdowns.

Since late 2020, the epidemiology and thus control of COVID-19 has changed due to two critical and opposing developments. The first is the emergence of novel variants of severe acute respiratory syndrome coronavirus 2 (SARS-COV-2) with higher transmissibility [8]. The second is the availability of several vaccines against SARS-CoV-2 [9], many of which have been in use since early 2021, 1 year after the disease began circulating globally.

It is unclear as yet whether herd immunity can be achieved with the current vaccination programme, as current vaccines are less effective at preventing transmission than preventing serious disease [10, 11]. Furthermore, until transmission is controlled globally, we are likely to continue to see the emergence of new variants, which may have higher transmission potential and increased severity of disease, including in the vaccinated and in vaccine-ineligible. Despite high coverage in wealthy nations [12], achieving high global vaccine coverage in most low- and middle-income countries will take years [13–15]. There is therefore a strong imperative to understand the continuing public health system requirements for detecting and controlling community transmission, including of variants of concern (VoCs), once planned vaccine programmes are completed and other public health control measures are lifted.

All confirmed cases of SARS-CoV-2 detected in the community in Australia in the period from 1 November 2020 to 30 June 2021 were linked to discrete outbreaks seeded from outside the country, and during this time Australia also had high rates of community testing. Hence, no cases of unknown origin were observed, meeting the criteria for considering that ongoing community transmission of SARS-CoV-2 in Australia was eliminated during this period [16]. All recorded outbreaks in this period have been linked through epidemiological investigation and genomic sequencing to international arrivals. Several resulted in local transmission in the community, all of which were subsequently contained, aside from outbreaks in Victoria and NSW (New South Wales) that commenced late in the study period and led to ongoing community transmission from July 2021 onwards.

The study period (1 November 2020 to 30 June 2021) therefore provides an ideal context in which to evaluate surveillance system performance as community transmission was controlled and case detection was close to complete [17]. The aim of this paper is therefore to define critical public health requirements for the ongoing management of COVID-19, including surveillance system requirements to support detection and control. This information informs current and future contexts of widespread vaccination, including approaches to international borders, and the emergence of VoCs. To address this aim, we:

1. Characterised and evaluated the performance of COVID-19 public health surveillance systems in detecting novel outbreaks of COVID-19 community transmission in the period from November 2020 to June 2021 in Australia
2. Used this evidence to define public health surveillance system requirements for current and future COVID-19 prevention, detection, and response.

## Methods

This paper describes a study evaluating the implementation of the COVID-19 surveillance system in Australia utilising publically available testing, case, and outbreak data. We applied the StaRI checklist for reporting of implementation studies when drafting our paper to ensure an accepted and standardised methodology and reporting of our study [18].

### Search strategy

We searched peer-reviewed literature and publicly available data to:

- Characterise the surveillance systems in place at the time of novel outbreaks (system characteristics, per capita testing rates, wastewater surveillance)
- Quantify and analyse the epidemiological characteristics of outbreaks occurring during this period (index cases, first diagnosed cases, total cases at time of first case being identified, links or transmission routes between cases, time from identification of the initial case to onset of infectivity in infected contacts)
- Conduct a public health analysis based on these results to establish requirements for COVID-19 detection and control under future transmission and response scenarios

### Data sources

The epidemiological data on outbreaks were collected from a range of government and media sources, including State and Federal government websites and online platforms of national and local public and commercial media outlets (see Additional file 1: Table of characteristics of community SARS-CoV-2 outbreaks, Australia, November 2020–June 2021),

The data source for state-level COVID-19 testing numbers was www.covid19data.com.au, a volunteer-run not-for-profit online platform [19]. The website verifies data with Federal, State, and Territory health departments before publishing. Population data used to calculate state-level COVID-19 testing rates were sourced from publicly available, government-managed data including the Australian Bureau of Statistics (ABS), Data NSW [20], and The Northern Territory Government Department of Industry, Tourism and Trade [21].

#### Surveillance system performance

Surveillance system performance was assessed based on sensitivity and timeliness in detecting introductions of COVID-19 into the community, calculated by comparing if and when an outbreak was first detected through surveillance, against information identified through subsequent epidemiological investigation, genomic testing, and linkages.

- *Sensitivity*: number of new outbreaks in study period first detected through surveillance/total number of new outbreaks in the study period.
- *Timeliness*: time between the seeding event linked to the primary (source) case and the date the index case was reported.

#### Public health analysis

Potential future SARS-CoV-2 transmission and response scenarios were developed based on the evolution of SARS-CoV-2 transmission and pathogenicity, alongside the development of public health control measures. The implications of surveillance system performance over the study period were then considered for these scenarios, including requirements for future surveillance and other key, non-surveillance-related public health control measures.

*Definitions*: see Additional file 2 for definitions of the following: testing rates, outbreak, cluster, index case, source case, and primary case.

## Results

### Components of Australia’s COVID-19 public health surveillance system

The different components of COVID-19 surveillance and screening used for the detection of new outbreaks of local transmission are described in the following sections.

#### Community-based surveillance for the disease in symptomatic individuals

A key component of Australia’s response has been surveillance for community transmission based on testing those with acute respiratory symptoms. The World Health Organization (WHO) recommended a syndromic screening definition for testing of “fever and cough” early in the outbreak [22]. Although often reported in patients with COVID-19, a substantial proportion of confirmed COVID-19 cases do not report these symptoms [23]. Australia has used a broader syndromic screening definition to target testing in the community since April 2020, which included any acute respiratory symptoms such as cough, sore throat, runny nose, cold symptoms, or flu-like symptoms [24].

Currently, the collection of most samples for community-based surveillance and testing in Australia is carried out in government-run testing centres and General Practice Respiratory Clinics. Between 30 [25] and 50% of individuals in the community with acute respiratory symptoms that meet the screening definition are being tested for COVID-19. Testing rates at an overall population level vary over time and by State, with marked increases in areas with confirmed outbreaks.

#### Genomic surveillance

During the study period, Australia aimed to fully sequence all cases of SARS-CoV-2 infection identified in the country including all cases in returned overseas travellers. AusTrakka, a platform for sharing of sequencing data, supports the Communicable Disease Genomic Network (CDGN), which includes all laboratories conducting SARS-CoV-2 genomics/diagnostics, to share sequencing data to support epidemiological outbreak investigation across the country. As of 7 May 2021, it is estimated that 58% of COVID-19 samples have been sequenced [26].

#### Screening of residents and staff linked to overseas traveller quarantine

Almost all international arrivals to Australia during the study period were required to undertake 2 weeks of mandatory supervised quarantine [27]. During this period, the policy required testing, regardless of symptoms, in the first 48 h and then again between days 10 and 12 of quarantine for SARS-CoV-2 infection [27], although exact protocols varied between States. Following incidents of transmission between travellers in quarantine facilities, national policy was changed in June 2021 to require returnees to undergo testing on day 16 (i.e. 2 days after release from quarantine) [28], although this was already the policy in several States prior to this. Routine, intensive testing of staff working in quarantine facilities commenced in January 2021; prior to this, testing was occurring, but less frequently.

#### Wastewater surveillance

Several states in Australia have ongoing wastewater testing programmes for COVID-19 which screen for viral fragments and VoCs. Positive samples are reported publicly by health departments to encourage those in the catchment areas to present for testing if they have symptoms. Positive wastewater detections are broadly classified as ‘expected’ (known positive or recovered cases in the catchment) or ‘unexpected’.

### Epidemiology of outbreaks, November 2020–June 2021 in Australia

Outbreaks and clusters initiated between 1 November 2020 and 30 June 2021 in Australia are described in Additional file 2: Characteristics of community SARS-CoV-2 outbreaks, Australia, November 2020–June 2021. A total of 30 outbreaks and clusters were reported in the study period, six in 2020 and 24 in 2021. Amongst these, 25 epidemiologically distinct outbreaks (i.e. no epidemiological link identified between that outbreak and others) and five distinct clusters (i.e. initially considered distinct but epidemiological link subsequently identified between that outbreak and others) were identified. All outbreaks were initially detected in major metropolitan areas, aside from one in the Northern Territory detected in a remote mine.

#### Index case

Of the 25 outbreaks, the index case was detected through community surveillance in 18 (72%) outbreaks and through quarantine–related screening of staff or residents in seven (28%). Of those detected through community surveillance, five were detected in individuals who had visited multiple states and/or territories while infectious and one was detected in a family member of a quarantine worker. Of those detected in quarantine residents or workers linked to quarantine, three were detected in residents after they left quarantine, and one was identified through testing of a symptomatic hospital healthcare worker who had treated patients referred from quarantine facilities.

Of the five clusters linked to outbreaks, all index cases were detected through community-based surveillance (including one in a healthcare worker), and only subsequently they were linked back to other outbreaks through previously undetected community transmission.

Of the 24 outbreaks or clusters that occurred in 2021, the index cases in 18 (75%) were detected through community surveillance (including two in healthcare workers) and in six (25%) through quarantine screening.

#### Source case

All outbreaks (and related clusters) aside from one were linked through genomic sequencing to breaches in quarantine facilities housing international travellers. The one exception was an outbreak in which the index (also primary) case was an international air crew limousine driver, and health authorities have suggested the most likely source is therefore also international. Sequencing also showed that a single overseas traveller was the common source for NSW-Croydon, VIC-Blackrock, and VIC-Vermont, all of which were seeded by the outbreak that commenced in NSW in December 2020 (Northern Beaches).

#### Primary case and early transmission links

Of the 30 outbreaks or clusters, an epidemiological link between the source case and the primary case (including those where both were the same) was identified in 17 (57%). The 13 outbreaks or clusters where no direct link was identified in publicly reported data included one in South Australia in November 2020; the NSW Northern Beaches outbreak and three related outbreaks or clusters with the same genomic sequence, one in NSW and two in Victoria; an outbreak in NSW in May 2021; and the outbreak suspected to be linked to air crew infecting a driver in NSW; and six outbreaks in Victoria in May and June 2021 all with the same genomic sequence as a returned overseas traveller from South Australian quarantine.

Of the 30 outbreaks and clusters, all except one included transmission in the community outside a quarantine setting. The one exception was an outbreak in a family of returned travellers infected while in quarantine. This family was detected as positive while in quarantine but subsequently found to have the same genomic sequence as the source case who was residing in the opposite room during their quarantine period.

#### Community mobility and interaction at time of disease incursion

Nineteen of the 25 outbreaks were seeded (i.e. the transmission event from the primary case) during periods of close to normal mobility (i.e. no masks in use, mass gatherings allowed, and no restrictions on the number of visitors in the home). Of the remaining outbreaks, four of these — although detected during periods in which restrictions were in place — are likely to have been seeded prior to implementation of those restrictions. This included the Croydon and Berala outbreaks detected in late December 2020 in NSW, which were identified during restrictions imposed on the 20th of December following the detection of the Northern Beaches outbreak; and VIC (Victoria) outbreaks in May and June (Arcare and West Melbourne), which were detected during restrictions imposed following the period when initial clusters were detected in Melbourne in late May. The outbreak in April in WA (Western Australia) commenced while mask wearing in public was in place following a previous outbreak; however, general community mobility and social interaction were not restricted.

All five clusters occurred during periods when there were other ongoing outbreaks and some level of restrictions.

### Surveillance system performance in detecting outbreaks

#### Community-based disease surveillance and outbreak detection

Initial identification of 21 (70%) of the 30 outbreaks/clusters was through testing of symptomatic community members, including two healthcare workers. For these 21 outbreaks/clusters, at the time of detection of the index case, the testing rate (percent of the total State population tested in the week preceding detection) was 2.07% on average. The average testing rate varied over time (1.68% in 2020 and 2.23% in 2021), and by the State in which the outbreak was detected.

In 2020, the testing rates in the week prior to detection of the outbreak in the five outbreaks/clusters detected through community surveillance ranged from 0.89 to 3.35% of the State population. The three outbreaks/clusters with rates at around 0.9%, in NSW, VIC, and SA, were detected in periods without any other ongoing outbreaks; one outbreak and one cluster in NSW both had testing rates of over 3%, and both were detected in periods when preceding outbreaks were still active.

In 2021, testing rates in the week prior to detection in the 20 outbreaks and four clusters ranged from 0.83% to over 4%. There were six outbreaks with testing rates over 3%, including five outbreaks in Victoria from May to June and the outbreak in NT (Northern Territory). The outbreak/clusters in Victoria in 2021 with testing rates of 1–2% were the Port Melbourne cluster and the Whittlesea cluster. These were both the initial outbreak/clusters in the May–June period, with the other outbreaks in this period occurring while these outbreaks were still active. There were two outbreaks/clusters with rates of less than 1%; these were the outbreaks in Brisbane in March and the Eastern suburbs outbreak in NSW in June.

#### Screening of quarantine residents and staff

Of the 24 outbreaks/clusters that occurred from January 2021 onwards, when intensive regular testing of quarantine staff commenced nationally, seven were identified through screening of quarantine residents or staff, including one case in a resident who had left the hotel quarantine and was tested after becoming symptomatic.

#### Genomic surveillance

All outbreaks/clusters were due to viruses with a novel genome sequence and not identified as circulating in the community prior to that outbreak/s. Of the 24 outbreaks/clusters in 2021, information on the strain of SAR-CoV-2 was available for 20. All were VoCs. Of these, seven were reported as the Delta variant, five as the Kappa variant, seven as the Alpha variant, and one was reported as a VoC without further specification.

#### Timeliness of detection

Of the 30 outbreak/clusters, data were publicly available on 17 to allow calculation of the time from the primary case until detection of the outbreak. The average delay was 4.9 days (4.7 for outbreaks and 5.7 for clusters), with 10 of the 17 outbreaks/clusters detected within 5 days and three detected after more than a week. These three included the NSW Berala cluster in January 2021 (10 days delay), the West Melbourne outbreak in June 2021 (detected after 9 days), and the Queensland hospital outbreak (10 days delay).

Of the 17 outbreaks/clusters with data, 11 were detected through community surveillance, four through quarantine, and two in healthcare workers who became symptomatic. The average delay for these groups was 5.7 days for community detection, 4 days for quarantine detection, and 3 days for healthcare worker detections.

#### Wastewater surveillance

All States/catchments reporting outbreaks/clusters in the period from 1 November 2020 to 17 June 2021 except for the NT had wastewater surveillance programmes during this period. Others that did not report outbreaks also had surveillance programmes (ACT, the Australia Capital Territory). All these locations reported positive wastewater samples within the study period (data not presented). Of those positive samples that were unexpected (i.e. no known positive or recovered cases in the catchment at the time of detection), one may have preceded the seeding event and therefore helped identify the outbreak through raising awareness and testing levels in the community (Coburg, VIC). The remaining unexpected detections were not linked to subsequently reported outbreaks.

### Surveillance system requirements for current and future control

The following section considers potential future SARS-CoV-2 transmission and response scenarios and analyses their surveillance system requirements. These future scenarios have been developed based on the evolution of SARS-CoV-2 transmission characteristics over the course of the pandemic thus far, alongside the evolution of public health control measures, in particular vaccination. See Additional file 3: Relationship between vaccination and other control measures, for consideration of the relationship between vaccination and other control measures on transmission.

The most important considerations when assessing novel variants will be their potential health system impacts. Any variant with increased severity including in those who are vaccinated and in those ineligible for vaccination, or with increased transmissibility and either similar or only marginally decreased severity to current variants, will result in increased health system burden. The majority of SARS-CoV-2 transmission occurs early in the course of the disease, prior to severe disease, and in those not at risk of severe disease [23, 29]. These characteristics select for strains with increased transmissibility but may not provide selective pressure against strains with higher mortality. The virus has already demonstrated the capacity to evade vaccine or disease-related immunity, and some evidence to date suggests that novel strains may also affect younger age groups and have increased mortality [8, 17, 29–32]. This is particularly important for those groups not eligible for vaccination, of which children are the biggest group [33]. There are reports of increasing mortality in children, including <5 years old, from those countries experiencing widespread transmission due to the Delta variant [30]. Availability of treatments such as monoclonal antibodies and antivirals may moderate the risk of variants being categorised as ‘of concern’ [34], if these treatments are effective and available to the whole of the population.

#### Community-based surveillance

Table 1 presents a public health analysis of the implications for community-based surveillance system requirements based on future scenarios. Irrespective of whether there is local endemic transmission, surveillance systems will need to maintain the capacity to detect variants with increased severity of disease. The key implication is that future surveillance systems will require a significant scale-up of genomic sequencing capacity to detect VoCs.

**Table 1:** Surveillance system requirements based on scenarios for COVID-19 epidemiology

Scenario	Key epidemiological characteristics/assumptions	Surveillance system capacity needed to meet requirements	Changes needed to the surveillance system
***A. While COVID-19 vaccination programme being rolled out***	Risk of substantial morbidity and mortality requires maintaining current community-based surveillance for disease and screening protocols for returned overseas travellers and staff in quarantine facilities, supported by genomic sequencing.	As per current requirements, i.e. as current surveillance system requirements are influenced by community acute respiratory illness level rather than SARS-CoV-2 rates [35], capacity requirements will be dictated by levels of ARI in the community.	Nil to maintain performance. However, if performance was improved, this is likely to make control of outbreaks, especially due to novel variants, easier to control. Performance could be further improved through improving testing rates (currently <50%) and timeliness in the symptomatic, particularly in communities at risk of low engagement in health interventions, and in high-risk occupations (e.g. healthcare workers).
***B (1) current vaccination programme completed; herd immunity achieved; current variants circulating***	Even if importations increase, transmission will be self-limiting in the general population, as the reproductive number will remain below 1 overall.	If herd immunity or even close to herd immunity is achieved through vaccination, SARS-CoV-2 levels will remain low, and community surveillance capacity will continue to be related to non-SARS-CoV-2 ARI rates.	Surveillance capacity will continue depending on background ARI rates (e.g. 4–6% of the population per week during the study period [25], likely to increase once social and mobility restrictions are fully lifted). Focus will need to include communities at risk of low uptake of health interventions, which may experience increased disease circulation and morbidity and mortality of both vaccine coverage and testing uptake are low.
***B (2) current vaccination programme is completed; herd immunity not achieved, current variants of concern circulating***	If herd immunity is not achieved through vaccination, and a decision is made not to utilise non-pharmaceutical interventions to a level that would eliminate transmission, this will mean endemic circulation of SARS-CoV-2, with the level of circulation dependent on the impact of the public health measures left in place.	It is likely that SARS-CoV-2 levels will eventually reach levels similar to other acute respiratory illnesses. If SARS-CoV-2 displaces other respiratory viruses [36], overall ARI levels will remain constant, and therefore also surveillance requirements. If COVID-19 infections are additive to the existing ARI burden, an increase in community surveillance capacity will be required.	If surveillance capacity needs to increase substantially, alternatives to PCR will be needed. For example, rapid antigen tests are already in widespread use in developed high-burden settings such as the UK and USA [37]. Although of lower sensitivity [37], overall performance will be similar or better than PCR if they facilitate more widespread and frequent screening. Prior analysis has shown that at a population prevalence of 3%, a test with 80% sensitivity that can test all the population exhaustively will miss 24% of cases, whereas test with 99.9% sensitivity, but which can only test a third of the population under surveillance, will miss 66% of cases in the population overall [35].
***C. Emergence of new variants of concern***	Given the characteristics of the virus to date, there is potential for new variants of concern to emerge that result in increased severity of disease, including in the vaccinated, as long as transmission is widespread globally [38].	The first level of surveillance for variants of concern relies on detecting SARS-CoV-2, and therefore the requirements outlined in the sections above apply. In addition, testing would need to include a greatly increased number of international arrivals. The second level of surveillance for early and effective detection of variants will rely on genomic sequencing of either all or an adequate proportion of all detected SARS-CoV-2 in the community and in international arrivals.	To achieve similar performance in detecting variants of concern as current capacity to detect SARS-CoV-2, sequencing capacity will need to be similar to current PCR-testing capacity, i.e. 1–2% of the population This equates to 200,000–400,000 samples per week nationally, given Australia’s population of ~21million. This is a >1000-fold increase from current sequencing levels.

*ARI* acute respiratory illness

#### Genomic sequencing

A population SARS-CoV-2 testing rate of 1–2% per week has been able to detect introductions of the virus early in transmission. To achieve similar performance in detecting VoC, capacity will be needed to sequence all SARS-CoV-2-positive samples, until or unless the number of positive samples reaches 1–2% of the population per week (as an estimated maximum) [39]. If positive SARS-CoV-2 samples reach this level, sequencing would need to shift to a randomly selected proportion of positive samples to maintain a rate of 1–2% population sequenced per week. This would require the capacity to sequence around 200,000–400,000 samples per week nationally, given Australia’s population of ~25 million. This is a 1000-fold increase from current levels, and it is unlikely to be achievable with whole genome sequencing. Development of technologies that can screen for, rapidly and at scale, specific mutations of particular concern will therefore be of great value (e.g. similar in principle to tools for screening for anti-tuberculosis drug resistance mutations, polymerase chain reaction (PCR) screening could be used to screen for S gene dropout in the Omicron variant of SARS-CoV-2) [40].

#### Screening in quarantine

Regular testing of all international arrivals, including residents and staff linked to quarantine facilities is an important component of the surveillance system. It gains even greater importance if the focus is early detection of imported variants, and genomic sequencing capacity cannot screen large numbers of community cases. The number of international arrivals (both resident and non-resident) to Australia in late 2019, prior to COVID-19-related border closures, was over 1.5 million per month [41]. Sequencing positive samples in this group alone would require a marked increase in capacity if positivity rates were to approximate prevalence in source countries.

#### Wastewater surveillance

Wastewater surveillance systems demonstrated limited utility over the study period in identifying and controlling novel outbreaks. Once levels of local transmission increase, there will be fewer catchments and periods where a positive sample is an unexpected finding. If the goal of surveillance shifts to identification of VoCs through sequencing of wastewater fragments [42], similar limitations in performance would apply. Given the limited performance in the study period, further evaluation of widespread wastewater surveillance must be done, including targeted surveillance of high-risk industrial and residential sites, to determine its added value against other COVID-19 surveillance strategies.

## Discussion

Once vaccine coverage rates are maximised, the priority for surveillance shifts from detection of SARS-CoV-2 to detection of SARS-CoV-2 VoCs associated with increased severity of disease. This surveillance will require ongoing investment to maintain current community-based disease surveillance systems and implement strategies for screening of returned travellers on a larger scale. Such surveillance systems will need to be linked to greatly increased genomic sequencing capacity if novel VoCs are to be detected early in transmission. Given Australia’s limited genomic sequencing capacity, there is an urgent need for investment in scaling up and decentralising, including through novel technologies capable of large volume throughput screening to detect mutations of concern.

Most novel incursions of SARS-CoV-2 into the Australian community have been detected through community-based surveillance for symptomatic disease, with the remainder detected through screening of returning overseas travellers and staff linked to quarantine facilities. Community-based symptomatic testing levels in the region of the outbreak were between 1 and 3% of the population tested in the week preceding detection of the outbreak, suggesting this level of testing will allow early detection of most community seeding or amplifying events. Outbreaks were detected, on average, within 5 days of the primary case. All outbreaks in 2021 were due to novel variants of concern. Although outbreaks due to the Delta variant of SARS-CoV-2 appear to be more transmissible and therefore more difficult to control, the surveillance system has still performed well in being able to detect them early.

The Australian national surveillance plan includes under goal 10 a range of indicators for timeliness of detecting individual cases [3]. Given that during the study period, a single case in the community in Australia represented an outbreak, these indicators would provide appropriate guidance against which to compare our findings. However, despite providing definitions of appropriate indicators of timeliness, the strategy does not define goal or targets for these indicators. A preliminary review of other Australian jurisdictions and internationally did not identify specific targets for timeliness either. This is an important gap that must be addressed by policymakers, and our study provides data to inform the development of appropriate and feasible targets in regard to detection of cases and outbreaks.

Wastewater surveillance was of limited utility in the study period. However, in the period following the study, from July to August 2021, there were two outbreaks in which detection of the outbreak was preceded by unexpected positive wastewater surveillance results [43–45]. Targeted screening of wastewater from high-risk settings (e.g. public housing towers [46]) has also been utilised in recent responses. Given the overall low sensitivity and specificity, detection will rely on community surveillance to rapidly identify cases if it is to be of utility in public health response, investing in maintaining community surveillance through high uptake of testing will continue to be critical.

As our analysis suggests, to have similar performance in detecting variants as Australia has had in detecting novel outbreaks of SARS-Co-V2, sequencing rates of up to 1000-fold magnitude higher than current requirements would be required. Prioritising sequencing to those samples linked to severe disease (hospital/ICU) would require less capacity. However previous research demonstrates that if surveillance were to rely on hospital rather than community-based case detection, the number of cases at the point of outbreak detection would be much higher, making outbreak control much more challenging [35]. A more effective strategy would be to prioritise sequencing to international arrivals, the most likely source of novel VoC introductions in Australia. This would require ongoing comprehensive SARS-CoV-2 testing of all arrivals. Even this alone would necessitate marked scale-up of sequencing capacity if international arrival numbers were to return to pre-COVID-19 levels.

Attempts of other high-income nations to detect SARS-CoV-2 variants highlight current constraints on the capacity of nations to conduct large-scale genomic sequencing. As of February 2021, the USA had sequenced around 96,000 (less than 1%) of its 27 million COVID-19-positive cases [47]. Experts attribute the country’s low sequencing rate to a lack of national coordination, poor surveillance planning, and inadequate investment in sequencing infrastructure and research [47]. In Canada, genomic surveillance has been a key component of the national surveillance strategy, with relatively greater success than the USA [48, 49]. An average of 5% of COVID-19-positive samples are being analysed across the country [50], with a target of sequencing 10% of positive samples [50]. This would bring its efforts closer to the UK, which has been lauded for its world-leading genomic sequencing capability [51]. As of 5 July 2021, the UK has conducted approximately 672,677 sequences out of 5,186,297 infected people (13.0%) in total [52].

However, even the UK’s level of sequencing is much lower than would be needed for Australia to conduct optimal surveillance for VoCs. Given the challenges faced by other developed countries in sequencing a high proportion of samples once the incidence of SARS-CoV-2 increases, it is unlikely that whole genome sequencing would be able to meet such requirements. It is therefore critical to implement genomic surveillance strategies that are effective in early detection of novel VoC introductions or emergence, to develop technologies that can screen for, rapidly and at scale, specific mutations of particular concern (e.g. immune escape mutations). Without such planning and adaptation for future scenarios of increased transmission, not only will it be impossible to sequence enough samples to meet surveillance goals, timeliness of sequencing will also be delayed, in turn delaying the identification of transmission links related to VoCs.

The Omicron variant of SARS-CoV-2 was first identified in late November 2021 globally and soon after designated a variant of concern by WHO [53]. By early December 2021, available data indicated increased transmissibility, including in vaccinated individuals, but impacts on the severity in vaccinated were unclear. The multiple mutations on the spike protein suggest there may be an immune escape and therefore a need for adapted vaccines. Even with Australia’s high levels of genomic sequencing, within 2 weeks of report of the first case, multiple jurisdictions were reporting Omicron cases, many with no known link to international travel or travellers [54]. This is likely to be due to levels of testing of overseas arrivals being around 40%. Retrospective sequencing of SARS-CoV-2-positive samples also identified further cases [55], indicating that higher rates of genomic surveillance may have detected community transmission of this variant earlier. Overall, the early information on the introduction of the Omicron variant supports the conclusions and recommendations made in this paper on the management of future variants based on data prior to its arrival.

A limitation of this study was that testing rates were calculated for States as a whole and therefore may not reflect the testing rates in the specific area where the outbreak occurred. Rates vary by geographical distance from testing sites. Given all outbreaks except one were detected in major metropolitan areas, which have higher testing rates than rural areas, State-level averages are likely to be an underestimate of testing in urban areas. However, testing rates also vary widely within urban areas based on the socio-economic characteristics of the catchment population. Testing data disaggregated by LGA were not publicly available for most States, but future analysis should be done to refine these estimates if such data becomes available.

Surveillance system requirements must be considered in the context of other public health measures to detect and control COVID-19 transmission. These are vaccine effectiveness, availability and uptake, outbreak response capacity (including effective implementation of social and mobility restrictions), and community engagement and partnership as the cross-cutting enabler of success in all areas. Additional file 4 provides a framework for future non-surveillance-related public health control measures when focus shifts to the management of VoCs.

As referred to previously, the impact of vaccination on transmission, and whether or not other measures are needed, depends in part on the effectiveness of the vaccine. See Additional file 4: Relationship between vaccination and other control measures. As has become apparent, knowledge is rapidly changing and evolving around the effectiveness of available vaccines even for current viral variants, such as the optimal number of doses for the primary schedule and the degree and timing of boosters to address waning of immunity [56]. The impact of vaccines is also related to vaccine availability, which is dependent on production, procurement, and distribution capacity. As seen in Australia in early 2020, limitations in all of these areas were the main barrier to vaccination [57, 58]. Additional file 4 assesses the requirements for ensuring that such barriers are minimised as much as possible in the event a novel vaccine is needed for a VoC. Targets are also critical, and a useful and ethical approach when assessing performance of vaccination programmes is to consider the highest coverage achievable with available vaccines and an effective community support and engagement strategy. Such a strategy would include offering every individual in the community for whom vaccination is not contraindicated their choice of approved vaccine, in a setting and at a time that is feasible and acceptable to them, alongside material support (paid leave, free health care, including primary practitioner support) for those likely to require such support and accessible, practical, and trusted messaging and communication. The target we propose in Additional file 4 is based on this approach. A key consideration is those groups at risk of lower engagement in health interventions, such as socially and economically disadvantaged groups. Past experience with vaccination suggests that the main cause for low uptake, including in these higher risk groups, is likely to be structural barriers to engagement [59, 60]. Therefore, rather than focussing on shifting behaviour in the small minority that are strongly vaccine resistant [61], a more useful and effective approach is to address structural barriers to engagement.

As well as surveillance, critical to Australia’s response has been outbreak response capacity [62], in particular the enumeration and management of cases and contacts efficiently enough to ensure that all transmission chains are controlled once initial seeding is identified through surveillance. As the outbreak evolved, innovations such as secondary contract tracing were needed in order to keep pace with the shortened serial interval of new variants [62]. Further innovations to increase the efficiency of outbreak response, including task shifting and automation where warranted, should be developed now in order to prepare for future scenarios (see Additional file 4). Border controls and social and mobility restrictions were also critical to effective control of SARS-CoV-2, prior to scale-up of testing, surveillance, and outbreak response capacity. These measures may again be critical if a variant emerges that exhibits the characteristics of concern discussed previously. For this reason, continued investments in understanding how these measures can be implemented efficiently and effectively while minimising secondary impacts are needed.

Finally, the most important factor underpinning success in prevention, detection, and control of current and future transmission, including our capacity to open society safely, will be how well governments support all sectors of the community. Investment in applied research on the structural barriers to engagement, and how these need to be addressed, is critical to ensuring success against COVID-19.

## Conclusions

Community-based surveillance for disease and routine screening of residents and staff in overseas quarantine facilities achieved early and comprehensive detection of new outbreaks of SARS-CoV-2 in the community in a period when local transmission had been eliminated in Australia and novel introductions through overseas arrivals were limited. Epidemiological investigation to identify transmission sources and link outbreak clusters has been greatly supported by comprehensive genomic sequencing.

Surveillance systems will need to emphasise the detection of VoCs through widespread genomic testing. Other measures to control VoCs that will need to be implemented alongside these surveillance strategies include maintaining and improving the efficiency of outbreak response and developing the capacity to rapidly engineer, manufacture, and distribute variant vaccines at scale, all underpinned by effective community partnerships. Finally, all the measures we have discussed are aimed at mitigating novel variants of concern, the main risk to the future control of COVID-19 in highly vaccinated populations. However, the only way to prevent their emergence is to support high vaccine coverage globally.

## Supplementary Information

**Additional file 1.** Table of characteristics of community SARS-CoV-2 outbreaks, Australia, November 2020-June 2021. A table summary of the characteristics of community SARS-CoV-2 outbreaks in Australia, November 2020-June 2021 including: location and dates, cases, outbreak or cluster, epi link source, date first notified, date primary case infectious, source, first identified, identified through community or quarantine, genomic variant, restrictions at time of detection, and state testing rate in preceding week.

**Additional file 2.** Definitions. A summary of definitions of terms including: testing rates, outbreak, cluster, index case, source case, and primary case.

**Additional file 3.** Relationship between vaccination and other control measures. Summary of the relationship between vaccination and other control measures on COVID-19 transmission.

**Additional file 4.** Public health response framework if VoCs with increased severity, including in vaccinated and/or vaccine ineligible, were to emerge. A table summary of a proposed public health response framework if VoCs with increased severity, including in vaccinated and/or vaccine ineligible, were to emerge.

## Data Availability

All data used are publicly available through the referenced sources.

## Abbreviations

ACT: The Australian Capital Territory
COVID-19: Coronavirus disease 2019
ICU: Intensive care unit
NSW: The state of New South Wales
NT: The Northern Territory
PCR: Polymerase chain reaction
SA: The state of South Australia
SARS-CoV-2: Severe acute respiratory syndrome coronavirus 2
VIC: The state of Victoria
VoCs: Variants of concern
WA: The state of Western Australia
