# Supplementary material for: Opening up safely: public health system requirements for ongoing COVID-19 management based on evaluation of Australia’s surveillance system performance
Source: BMC Med. 2022 Apr 15;20:157. doi: 10.1186/s12916-022-02344-x (PMC9010199; doi:10.1186/s12916-022-02344-x)
Supplement: Supplementary file 2 — Additional file 2. Definitions. A summary of definitions of terms including: testing rates, outbreak, cluster, index case, source case, and primary case. [file 12916_2022_2344_MOESM2_ESM.docx]

## Additional File 2.

#### Definitions

***Testing rates*** were defined as the proportion of the total population in that State tested for Sars-CoV-2. These were calculated for the week preceding the detection of the outbreak, for the State in which the outbreak occurred.

***Outbreak definition:*** Outbreaks of COVID-19 included in this analysis included any in which one or more cases were due to community (locally-acquired) transmission within the specified time period from 1^st^ November 2020 to 30^th^ June 2021. Community transmission included any local transmission in the community, as well as any cases in quarantine or health workers in direct contact with international travellers. It also included international travellers who were infected while in quarantine in Australia, but excluded those international travellers who were infected prior to entering quarantine or who were infected by household/family contacts with whom they were residing while in quarantine. Each individual outbreak was defined as all cases epidemiologically linked to each other. If epidemiological links were not identified between distinct clusters of cases, these clusters were still considered different outbreaks even if a common source in a returned overseas traveller was identified through genomic sequencing.

***Cluster definition***: A cluster was defined as a distinct group of cases initially identified as an outbreak but which was eventually linked to another outbreak via epidemiological evidence and therefore did not meet the above outbreak definition. However, as these clusters were included as they provide useful information on surveillance system performance in detecting transmission.

***Index case:*** The index case was defined as the first reported case identified in that outbreak.

***Source case:*** The source case was defined as the case with the earliest date of symptom onset and/or infectivity that could be linked to that outbreak through genomic evidence.

***Primary case:*** The primary case was defined as the case with the earliest date of symptom onset and/or infectivity that could be epidemiologically linked to that outbreak.

(Note: in general, the epidemiological terms ‘primary’ and ‘source’ case are used interchangeably as they are the same individual. However, in this paper we have differentiated these 2 terms and adopted slightly different definitions, as described above, in order to account for the fact that the true primary /source case, (which was always an international arrival) was often not linked epidemiologically, despite being linked through genomics. By differentiating, we were able to better describe the characteristics of the outbreaks and their detection).
