# Supplementary material for: Opening up safely: public health system requirements for ongoing COVID-19 management based on evaluation of Australia’s surveillance system performance
Source: BMC Med. 2022 Apr 15;20:157. doi: 10.1186/s12916-022-02344-x (PMC9010199; doi:10.1186/s12916-022-02344-x)
Supplement: Supplementary file 3 — Additional file 3. Relationship between vaccination and other control measures. Summary of the relationship between vaccination and other control measures on COVID-19 transmission. [file 12916_2022_2344_MOESM3_ESM.docx]

## Additional File 3.

***Relationship between vaccination and other control measures***:

Ignoring differences by age and different vaccine characteristics, we achieve herd immunity if V*VE > 1 - 1/R, where V = proportion vaccinated; VE = vaccine efficacy; R = reproductive number. Even if herd immunity is not achieved, control of transmission can still be maintained if any increase in transmission potential, for example through less stringent restrictions, is offset by gains in control made by increased vaccine coverage (i.e., such that the overall reproductive number remains below 1). Once the vaccination program has achieved its targets, public health responses will be based on the post-vaccination effective reproduction number in the community, with the relationship between different measures continuing as defined above. This may also include booster vaccines to address waning immunity against existing strains.^1^ However, irrespective of whether herd immunity is achieved or not, (and if not achieved, whether control of current SARS-CoV-2 strains is aimed for or not), the capacity to prevent, detect and control the spread of novel VoCs will need to be ongoing while transmission is widespread globally.^2^

**References**

1. Associated Press. Israel to offer Pfizer Covid booster shots to people over 60: The Guardian; [updated 30 July 2021. Available from: <https://www.theguardian.com/world/2021/jul/30/israel-to-offer-pfizer-covid-booster-shots-to-people-over-60>.
2. World Health Organization. COVID-19 Weekly Epidemiological Update. 2021 22 June 2021.
