# Supplementary material for: Opening up safely: public health system requirements for ongoing COVID-19 management based on evaluation of Australia’s surveillance system performance
Source: BMC Med. 2022 Apr 15;20:157. doi: 10.1186/s12916-022-02344-x (PMC9010199; doi:10.1186/s12916-022-02344-x)
Supplement: Supplementary file 4 — Additional file 4. Public health response framework if VoCs with increased severity, including in vaccinated and/or vaccine ineligible, were to emerge. A table summary of a proposed public health response framework if VoCs with increased severity, including in vaccinated and/or vaccine ineligible, were to emerge. [file 12916_2022_2344_MOESM4_ESM.docx]

#### Additional File 4:

***Public health response framework if VoCs with increased severity, including in vaccinated and/or vaccine ineligible, were to emerge***

|  | **Vaccine development** | **Vaccine delivery** | **International border management & quarantine** | **Outbreak response** | **Social and mobility restrictions** |
| --- | --- | --- | --- | --- | --- |
| **Goal** | Rapid development and manufacture of vaccines for emerging VoC | Rapid delivery of boosters for emerging VoC | Prevention of entry of VoC | Rapid control of VoC | Prevention of widespread morbidity and mortality due to VoC |
| **Key considerations** | Vaccines for future variants that show increased mortality amongst the vaccinated / vaccine ineligible can only be developed after detection of the variant.  Local vaccine development and large-scale manufacture has been the most effective strategy for meeting vaccine needs to date^1^. Variant vaccines will also need to not be inactivated by immunity to prior vaccine vectors. mRNA vaccine platforms are most likely to meet these requirements. | Rapid vaccine distribution at scale will be required for VoC.i Rapid mass distribution capacity has not been tested to date given the slow supply and initial shortages of vaccines.^[[1]](#footnote-1)^ Systems will continue to rely on general practice as well as mass vaccination hubs^[[2]](#footnote-2)^, and will need to include ultra-low temperature cold chain capacity,^[[3]](#footnote-3)^ which may be a characteristic of future variant vaccines.  Target vaccination coverage rates proposed for developed countries are generally at or above 70% of the overall population.  Experience from other settings suggests that achieving this vaccine coverage will rely on a range of strategies that can take vaccines close to every sector of the community^[[4]](#footnote-4)^. | Self-contained cabin-style accommodation with natural ventilation has to date been the most effective form of quarantine, and increased capacity in such facilities is planned.^[[5]](#footnote-5)^ However, as borders open, it will not be possible or necessary to accommodate all international arrivals in such facilities, and alternative measures will need to be considered for low-risk arrivals (e.g. monitored home quarantine as is in use in Singapore and Taiwan,^[[6]](#footnote-6)^ and/or testing).  Given the frequency of quarantine breaches, and the huge impact of such breaches, further investment in strengthening quarantine facility structures, as well as more frequent testing of staff and arrivals in such facilities, would be appropriate | Control of VoC community transmission will rely on the same outbreak response measures used to date (case and contact management, infection control etc).  Improvements in outbreak management have been achieved through increased workforce, computerized systems for data management, more sensitive definitions of contacts, management of secondary and casual contacts, computerized registration at venues and gatherings (QR codes) managed centrally by governments, and interoperability of systems nationally | Stringent lockdowns will not have a place in control, except for VoC. If implemented for VoC in future, the severity and duration of restrictions will depend on how long it takes to develop and distribute effective variant vaccines. Given the cost of restrictions is much larger than establishing mass vaccine manufacturing and distribution, these are sound economic as well as public health investments.^[[7]](#footnote-7)^  Minimise the following risk factors for outbreaks that overwhelm other public health control measures: delay in detection of outbreak; lack of access, information and mistrust of public health authorities in outbreak community; exposure sites with large numbers of high-risk close contacts; novel variants with increased transmissibility |
| **Key performance indicator** | Production capacity targets based on populations in the country of manufacture as well as neighbouring countries without their own production capacity, receiving rapid, adequate supplies. | All of the population, including subgroups at risk of lower engagement in health interventions, is aware of a vaccination site within 30 minutes’ travel of their place of residence and/or work. Accurate information on the benefits and risks of vaccination has been conveyed to them by an information source they trust. | Number and rate (per positive traveller) of breaches for travellers returning from high-risk locations (i.e. settings with VoC in circulation). | Contact tracing – including its timeliness and efficiency –is optimal and incudes secondary contact tracing. Recommendations from reviews of outbreaks in aged care, health care and other high-risk settings have been implemented.^[[8]](#footnote-8)^ | Stringent restrictions implemented as soon as VoC are detected and there are indicators that other control measures will be inadequate. |
| ***Community partnership and engagement*** | Community partnership, support and engagement is critical to every aspect of successful COVID-19 management, including achieving high testing rates in symptomatic individuals, compliance with isolation and quarantine, and vaccine uptake. Equally, even if there is an evidence-based pathway for shifting out of non-pharmaceutical control measures, unless the community is engaged in this decision making process, there is the potential for reduced community confidence and lower engagement in perceived non-essential/ higher-risk activities (e.g. discretionary consumer spending).^[[9]](#footnote-9)^ Addressing this will necessitate a staged reopening^[[10]](#footnote-10)^ that gives the community confidence at each step that adverse health consequences are unlikely for themselves and others, and that a return to restrictions is not a policy consideration. In practice, this would, for example, mean that communities and their trusted community partners design and lead response strategies, and that these strategies have embedded in them real-time systems for identifying structural barriers to uptake, and resources and pathways for addressing those barriers. There is increasing recognition in Australia that this is a critical need,^[[11]](#footnote-11)^ but the expertise on how to do this, and therefore the models for effective community partnership and engagement are still sub-optimal, particularly for the hardest-to-reach groups. Lessons can and must be learnt from sectors that have invested in building such expertise: e.g. Indigenous community-controlled health, applied sociological and anthropological expertise in developing countries from epidemics such as Ebola.^[[12]](#footnote-12)^ | | | | |

1. MacMillan J. Local production of mRNA COVID-19 vaccines could take up to four years, government says. ABC News. 3 Jun 2021, <https://www.abc.net.au/news/2021-06-03/mrna-covid-19-vaccines-local-production-timeline/100187642>; Butler J. Why doesn’t Australia have mRNA vaccine factories yet? The New Daily. Apr 19, 2021, https://thenewdaily.com.au/news/2021/04/19/mrna-vaccine-factory-australila/ [↑](#footnote-ref-1)
2. Australian Government. Australian COVID-19 Vaccination Policy, 13 November 2020, Last Updated 17 February 2021, https://www.health.gov.au/sites/default/files/documents/2020/12/covid-19-vaccination-australian-covid-19-vaccination-policy.pdf [↑](#footnote-ref-2)
3. Holm MR. Critical aspects of packaging, storage, preparation, and administration of mRNA and adenovirus-vectored COVID-19 vaccines for optimal efficacy. *Vaccine* 2021; **39**(3): 457-9. [↑](#footnote-ref-3)
4. Centers for Disease Control and Prevention. COVID-19 Vaccinations in the United States. 11 July 2021. https://covid.cdc.gov/covid-data-tracker/#vaccinations. [↑](#footnote-ref-4)
5. Australian Government Department of Health. National Review of Hotel Quarantine, 2020, <https://www.health.gov.au/sites/default/files/documents/2020/10/national-review-of-hotel-quarantine.pdf>; Northern Territory Government. Howard Springs moving forward with expansion. 30 April 2021. https://coronavirus.nt.gov.au/updates/items/2021-04-30-howard-springs-moving-forward-with-expansion. [↑](#footnote-ref-5)
6. Taiwan Centers for Disease Control. COVID-19 Coronavirus disease 2019 FAQs. 2021/6/30. <https://www.cdc.gov.tw/En/Category/QAPage/SbkmnM5v0OwdDMjJ2tI_xw>; Singapore Government. Stay Home Notice (SHN). 11 Jul 2021. https://safetravel.ica.gov.sg/health/shn. [↑](#footnote-ref-6)
7. Australian Academy of Science. 2021-22 Pre-Budget Submission, https://www.science.org.au/files/userfiles/support/submissions/2021/prebudget21-22-aas.pdf [↑](#footnote-ref-7)
8. Experts criticise Australia's aged care failings over COVID-19. *The Lancet* 2020; **396**(10259): 1322-3; Boseley M. Worksafe investigates coronavirus cluster at Cedar Meats as workers speak out. The Guardian. 13 May 2020; The Australian Medical Association. Hotel quarantine failures show need for action. 11 Feb 2021. https://www.ama.com.au/ama-rounds/12-february-2021/articles/hotel-quarantine-failures-show-need-action. [↑](#footnote-ref-8)
9. Wade M. ‘More sophisticated’: The pandemic has changed how Australians are spending money. The Sydney Morning Herald. 17 February 2021. <https://www.smh.com.au/business/the-economy/more-sophisticated-the-pandemic-has-changed-how-australians-are-spending-money-20210216-p572yt.html>; Jin X, Zhao Y, Song W, Zhao T. Save for Safe: Effect of COVID-19 Pandemic on Consumers' Saving and Spending Behavior in China. *Front Psychol* 2021; **12**: 636859. [↑](#footnote-ref-9)
10. Beginning with low risk arrivals (vaccinated individuals from low risk countries, continued stringent screening and quarantine pre and post arrival) with the highest individual social/economic benefits (e.g. skilled migrants, postgraduate students etc). [↑](#footnote-ref-10)
11. Maher R, Murphet B. Community engagement in Australia’s COVID-19 communications response: learning lessons from the humanitarian sector. Media International Australia 2020; 177(1): 113-8. [↑](#footnote-ref-11)
12. Gilmore B, Ndejjo R, Tchetchia A, et al. Community engagement for COVID-19 prevention and control: a rapid evidence synthesis. 2020; 5(10): e003188 [↑](#footnote-ref-12)
